# Identification of Lysine Acetylation Sites on MERS-CoV Replicase pp1ab

**DOI:** 10.1074/mcp.RA119.001897

**Published:** 2020-11-23

**Authors:** Lin Zhu, Sin-Yee Fung, Guangshan Xie, Lok-Yin Roy Wong, Dong-Yan Jin, Zongwei Cai

**Affiliations:** 1 State Key Laboratory of Environmental and Biological Analysis, Department of Chemistry, Hong Kong Baptist University, Kowloon Tong, Hong Kong; 2 School of Biomedical Sciences, The University of Hong Kong, Pok Fu Lam, Hong Kong

**Keywords:** Acetylation, post-translational modifications, viruses, immunoaffinity, bioinformatics, MERS-CoV

## Abstract

MERS is a life-threatening disease and MERS-CoV has the potential to cause the next pandemic. Protein acetylation is known to play a crucial role in host response to viral infection. Acetylation of viral proteins encoded by other RNA viruses have been reported to affect viral replication. It is therefore of interest to see whether MERS-CoV proteins are also acetylated. Viral proteins obtained from infected cells were trypsin-digested into peptides. Acetylated peptides were enriched by immunoprecipitation and subject to nano-LC-Orbitrap analysis. Bioinformatic analysis was performed to assess the conservation level of identified acetylation sites and to predict the upstream regulatory factors. A total of 12 acetylation sites were identified from 7 peptides, which all belong to the replicase polyprotein pp1ab. All identified acetylation sites were found to be highly conserved across MERS-CoV sequences in NCBI database. Upstream factors, including deacetylases of the SIRT1 and HDAC families as well as acetyltransferases of the TIP60 family, were predicted to be responsible for regulating the acetylation events identified. Western blotting confirms that acetylation events indeed occur on pp1ab protein by expressing NSP4 in HEK293 cells. Acetylation events on MERS-CoV viral protein pp1ab were identified for the first time, which indicate that MERS-CoV might use the host acetylation machinery to regulate its enzyme activity and to achieve optimal replication. Upstream factors were predicted, which might facilitate further analysis of the regulatory mechanism of MERS-CoV replication.

## Teaser

MERS is a life-threatening coronavirus and its mechanism is not clearly understood. In this study, acetylation events on MERS-CoV viral protein pp1ab were identified for the first time, which indicate that MERS-CoV might use the host acetylation machinery to regulate its enzyme activity and to achieve optimal replication. Upstream factors were predicted, which might facilitate further analysis of the regulatory mechanism of MERS-CoV replication.

## Graphical Abstract

**Figure:**
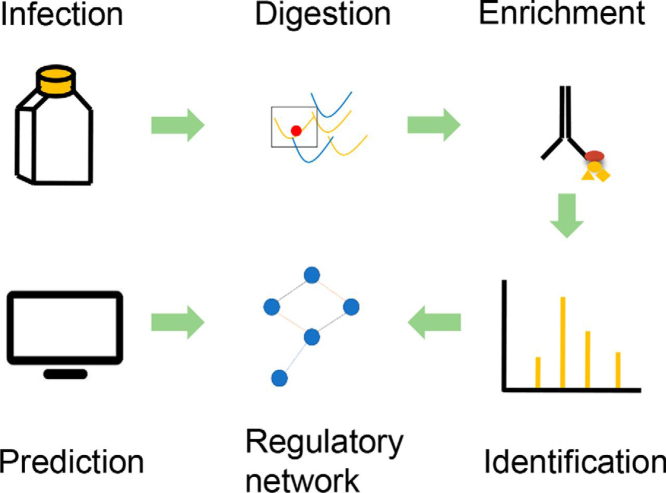

**Highlights**

• Acetylation sites on MERS-CoV protein pp1ab were reported for the first time.
• Sirt1 was predicted as upstream factor of identified acetylation events.

First isolated in 2012 (1), Middle East respiratory syndrome coronavirus (MERS-CoV) is an emerging virus leading to severe and highly lethal respiratory diseases. According to WHO, MERS-CoV has a high case fatality of over 35% among more than 2500 cases reported from 27 countries worldwide (2). MERS-CoV is a potential pandemic agent as person-to-person transmission has been observed, particularly within health care settings (3). Therapeutic options are limited and unspecific (4). More importantly, unlike the epidemic of SARS-CoV that quickly faded off, the MERS epidemic has persisted over years with no signs of subsiding (5). It is currently known that the clinical outcome might be dictated by the abilities of the virus to infect a wide range of DPP4-expressing cells, to induce dysregulation of cytokines and to evade host innate immune response (4). However, detailed molecular mechanisms for transmission and virus-host interaction remain poorly understood.

Post-translational modifications (PTMs) generally refer to reversible addition of a functional group covalently to specific amino acid residue(s) on a protein (6). PTMs provide additional levels of regulation that could respond simultaneously to external stimuli. Among more than 400 different PTMs discovered so far, lysine acetylation is one of the most crucial types. Protein acetylation can control protein location, stability, and enzymatic activity (7). Particularly, accumulating evidence suggests that acetylation serves crucial roles in regulating both host response to viral infection and viral replication process (8). Transcriptional activity of NF-κB complex is regulated by acetylation on its subunits (9). Viral DNA sensor IFI16 requires acetylation to translocate to the nucleus to initiate innate immune response (10). Viruses can perturb host anti-viral response by inhibiting p53 acetylation (11).

Emerging evidence shows that viral proteins are subject to acetylation by host factors. Acetylation of HIV-1 transactivator Tat is required for its transcriptional activity (12); whereas acetylation of influenza A ribonucleoprotein NP was shown to be crucial for normal viral replication and packaging (13). Considering that a viral protein might serve multiple functions during its replication cycle, acetylation provides a variety of protein isoforms that probably fit the needs. Particularly, acetylation occurs on different positions or functional domains to generate different protein isoforms with distinct functional activities. Moreover, to identify PTM sites on viral proteins provides crucial information for developing antiviral drugs (8). However, no acetylation site has been reported for any MERS-CoV viral protein so far. In this study, we first reported multiple acetylation sites on MERS-CoV replicase pp1ab, suggesting a potential link of protein acetylation to the regulation of MERS-CoV replication.

## EXPERIMENTAL PROCEDURES

##### Experimental Design and Statistical Rationale

Three biological repeats of sample infected with MERS-CoV at 1 or 5 M.O.I. have been performed. As the focus of study is to search for potential acetylation sites on MERS-CoV proteins, no control or randomization was performed in the study. No quantitation analysis has been performed and therefore no statistic method was used in the study. All data and search result in msf format has been deposit to Harvard Dataverse and available for reviewing at following address: https://doi.org/10.7910/DVN/MTDOG7.

##### Virus Preparation

MERS -CoV was a gift from Dr. Ron Fouchier (Erasmus Medical Center, Rotterdam, the Netherlands) and cultured in VeroE6 cells in serum free DMEM. Viruses were produced by transfection of the infectious clones into Vero-E6 cells according to previous described (14) The experiment was carried out in a biosafety level 3 laboratory and strictly followed the disinfection protocol that boiling protein sample in 10% SDS.

##### In-solution Trypsin Digestion

In solution digestion was performed as previously described (15) with minor modifications. Briefly, the disinfected protein samples were firstly precipitated by 4 times volume of acetone, followed by resuspended with 8 m Urea, 50 mm Ammonium bicarbonate, 1% RapiGest SF Surfactant (Waters) to solubilize the sample. The protein sample was then reduced by 5 mm DTT and alkylated by 15 mm IAA. Sequencing grade trypsin (thermo scientific) was used at 1:50 enzyme/protein ratio to digest the sample at 37 °C overnight. The surfactant was removed by adding 200 mm HCl and centrifuged at 20,000 × *g* for 10 min.

##### Acetylated Peptide Enrichment

The resulting peptides were then used for acetylated peptide enrichment. Acetyl lysine antibody (Immunechem, Canada) conjugated on agarose beads was used to immunoprecipitated the acetylated peptides (16). Briefly, the tryptic digested peptide mixture was resuspend in NETNA buffer (50 mm HEPES, 100 mm NaCl, 1 mm EDTA, 0.5% NP-40 and 10% Acetonitrile) and incubated with the acetyl lysine antibody beads overnight. The beads was then washed with same buffer for three times, before eluted with 5% TFA in 10% Acetonitrile. The eluted acetylated peptides were freeze-dried in a freeze dryer and resuspend in 0.1% formic acid for LC-MS analysis.

##### LC-MS/MS Analysis

Peptides were loaded on a self-pack C18 analytical reverse phase column (ID 75 μm × 15 cm, 200Å, 3 μm particles) at flow rate of 300 nL/min and a 75 min LC gradient of 8% to 28% ACN in 0.1% FA was used. Orbitrap fusion tribrid (Thermo-Fisher) MS machine was used to analyze the peptide sample in a data-dependent acquisition mode, with 120,000 resolution at MS1 scan and 30,000 at MS2 (FWHM at *m*/*z* 400); cycle time 3 s, AGC target for MS2 is 50000, maximum injection time 60 ms, HCD collision energy set at 35%.

##### Database Search

The resulting Raw data file was then analyzed with Sequest-HT (2013) integrated in software Proteome Discoverer software (1.4.1.14, ThermoFisher) against Uniprot MERS-CoV database, which was composed of 10 reviewed sequences from Swiss-Prot on the Uniprot website released on Nov 18^th^, 2017. Trypsin is set as digestion enzyme and maximum allowed miss cleavage is 2. Precursor mass tolerance set at 20 ppm, fragment mass tolerance is 0.02 Da, carbamidomethyl on cysteine as fix modification and acetylation on lysine is variable modification. FDR is set at 0.01. The Protein FDR Validator node now bases its validation on protein scores calculated from the posterior error probability (PEP) values of the peptides if these values are available. A high confidence level (1% FDR) was set to filter out peptides identified with lower confidence based on the Proteome Discoverer software.

For identified acetyl-lysine carrying peptides, manual validation has been carried out to confirm the acetylation site assignment as well as the identification of peptide. The validation is based on checking the fragmentation pattern of the peptides.

##### Bioinformatic Analysis

A total of 540 sequences derived from MERS-CoV virus pp1ab were extracted from NCBI database, and sequence alignment was then performed by online tool Cluster Omega (http://www.ebi.ac.uk/Tools/msa/clustalo/) (17). KAT-specific acetylation and SIRT1 substrate site prediction with pp1ab sequence was done by a web-based server based on ASEB method (18, 19) (http://bioinfo.bjmu.edu.cn/huac/predict_p/). Briefly, the method focused on searching potential KAc sites that have a similar sequence with known ones. The calculation was based on the 17 amino acid peptides flanking the hypothetic KAc site. At first step, similarity scores were obtained between the potential KAc peptide and known database list. An enrichment score was then calculated as the sum score of all similarity scores for each known peptides in the list. Then the significance of enrichment was estimated against a random peptide set including 9999 peptide sequences. Histone acetyltransferases CBP/p300, GCN5/PCAF and MYST and histone deacetylases class I HDAC and SIRT1 were tested by the web default setting one by one and significant sites were picked out at top 4%, 1%, 1%, 2%, and 1%, respectively.

## RESULTS

To look for lysine acetylation sites on MERS-CoV viral proteins under physiologically more relevant conditions, infected cells were used instead of cells over-expressing a single viral protein. Briefly, Vero-E6 cells were infected with MERS-CoV at a Multiplicity of infection (M.O.I.) of 1 or 5 respectively for 48 h, before the viral proteins were harvested *via* ultrahigh speed centrifugation followed by boiling in 10% SDS. The resulting and disinfected viral protein samples were pooled together and subject to standard in-solution digestion with some modifications. As lysine acetylation was a relatively rare event, acetylated peptides were enriched from trypsin-digested peptides by immunoprecipitation with anti-acetyl-lysine antibody, and then analyzed by orbitrap MS machine coupling to a nano-UPLC system.

**Fig. 1:**
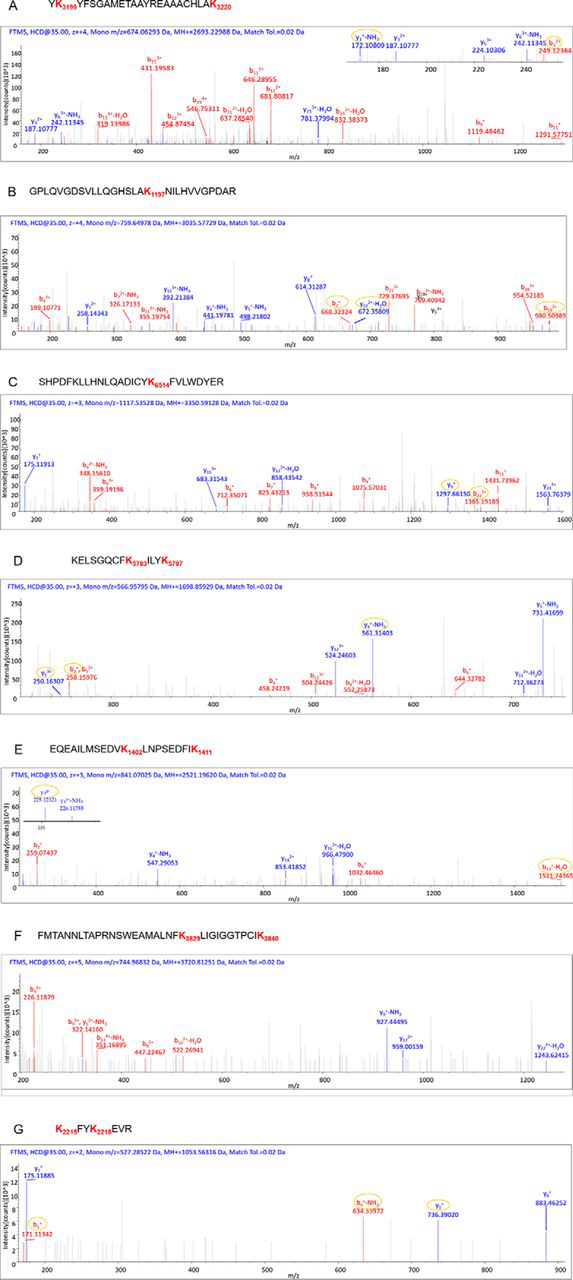
**MS2 spectrum of identified acetylated peptides.** Representative spectrum of identified spectrums of acetyl-lysine containing peptides from pp1ab protein (accession number: K9N7C7). A: YKYFSGAMETAAYREAAACHLAK; B: GPLQVGDSVLLQGHSLAKNILHVVGPDAR; C: SHPDFKLLHNLQADICYKFVLWDYER; D: KELSGQCFKILYK; E: EQEAILMSEDVKLNPSEDFIK; F: FMTANNLTAPRNSWEAMALNFKLIGIGGTPCIK; G: KFYKEVR. Acetylation sites are marked in red in the peptide sequence and the amino acid site number are marked in subtitle as well. For each peptide spectrum, key fragment ions are marked with yellow circle.

**Fig. 2:**
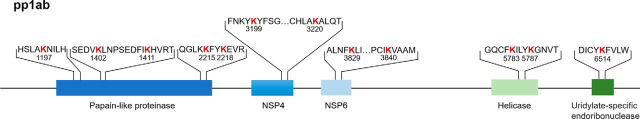
Distribution of acetylation sites on ORFs of pp1ab.

A total of 39 peptides from 7 proteins were identified, among which 21 were reported to be acetylated (Supplemental Table 1). The enrichment efficiency of acetylated-lysine peptides was therefore 53.2%. After manually check the spectrum to remove peptides with ambiguous acetylation sites or poorly ionized spectrum, seven acetylated peptides with twelve acetylation sites were reported (Fig. 1 and Table I). All acetylation sites identified are located on polyprotein pp1ab, a multifunctional protein further processed into 15 individual products, each of which carries different functions in MERS-CoV replication (Fig. 2). Although many acetylation sites were in the papain-like proteinase region at the N terminus of pp1ab, acetylation sites were also found in helicase, uridylate-specific endoribonuclease, nsp4 and nsp6. Multiple sequence alignment of 540 entries of MERS-CoV pp1ab sequence from NCBI database showed that all acetyl-carrying lysine sites are highly conserved among MERS-CoV viruses (Table II), suggesting a potential important role of these amino acid residues in virus growth or replication. As other studies have demonstrated that protease activity could be regulated by deacetylase inhibitors (20), and that acetylation on helicase or endonuclease could promote DNA stability (21), MERS-CoV might also utilize the host acetylation machinery to regulate its own proteinase activity to achieve optimal replication.

**Table I:** Identified acetyl-lysine carrying peptides in pp1ab (Accession no.: K9N7C7)

Peptide Sequence	Monoisotopic (*m*/*z*)	Error (ppm)	Sequence start/end	Lys-acetylation site(s)	PEP	M (Da)
YKYFSGAMETAAYREAAAcHLAK	674.063	−7.01	3198–3220	3199, 3220	0.0628	2692.222
GPLQVGDSVLLQGHSLAKNILHVVGPDAR	759.650	−12.81	1180–1208	1197	0.159	3034.569
SHPDFKLLHNLQADICYKFVLWDYER	1117.535	−11.68	6497–6522	6514	0.41	3349.583
KELSGQCFKILYK	566.958	−10.53	5775–5787	5783, 5787	0.305	1697.851
EQEAILMSEDVKLNPSEDFIK	841.070	2.33	1391–1411	1402, 1411	0.289	2520.188
FMTANNLTAPRNSWEAMALNFKLIGIGGTPCIK	744.968	−15.47	3808–3840	3829, 3840	0.108	3719.805
KFYKEVR	527.285	−9.14	2215–2221	2215, 2218	0.243	1052.555

**Table II:** Evolutionally conservative and location of acetylation sites identified

Site	% of conservation^a^	ORF
K1197	100%	Papain-like proteinase
K1402	99%	Papain-like proteinase
K1411	100%	Papain-like proteinase
K2215	96.30%	Papain-like proteinase
K2218	99.80%	Papain-like proteinase
K3199	99.80%	Non-structural protein 4
K3220	99.40%	Non-structural protein 4
K3829	99.60%	Non-structural protein 6
K3840	99.80%	Non-structural protein 6
K5783	100%	Helicase
K5787	99.80%	Helicase
K6514	98.10%	Uridylate-specific endoribonuclease

a Generated from 540 Mers CoV replicase 1ab sequences fetched from NCBI database (on Oct 28th 2019).

To further understand how the acetylation events might affect viral functions, it would be of interest to investigate the upstream factors that regulate lysine acetylation. Motif analysis of flanking amino acid sequences of the identified acetylation sites did not reveal a significant conserved motif, which suggested the involvement of multiple acetyltransferases and deacetylases in the process.

We therefore used a prediction-based bioinformatic method to analyze which lysine residues on pp1ab could be acetylated in theory, then matched the prediction list with our MS-identified acetyl-lysine sites. Histone acetyltransferases of CBP/p300, GCN5/PCAF and MYST families as well as deacetylases class I HDAC and SIRT1 were chosen to predict their corresponding regulatory sites on pp1ab, based on their known substrate sequences (supplemental Table S2). Interestingly, two identified acetylated lysine sites were indeed found to overlap with the predicted list. K3220 was predicted to be a substrate of SIRT1 deacetylase, whereas K3840 was predicted to be acetylated by TIP60 family, and deacetylated by HDAC family (Table III). The bioinformatic analysis suggested that these acetylases and deacetylases are likely employed by the MERS-CoV to modify its own protein to achieve optimal growth.

**Table III:** Predicted upstream KAT/deacetylase of acetylation sites

KAT/Deacetylase	Site	Sequence	*p* value
SIRT1	3220	EAAACHLAKALQTYSET	0.0232
HDAC1/HDAC2/HDAC3	3840	GIGGTPCIKVAAMQSKL	0.046
TIP60/MYST1/2/3/4	3840	GIGGTPCIKVAAMQSKL	0.0615

**Fig. 3:**
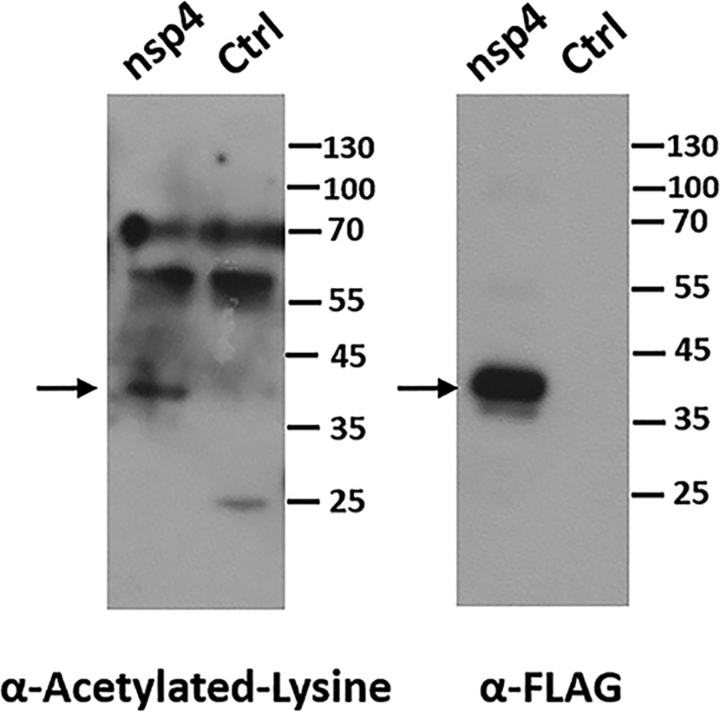
**Immunoblotting with pan-acetyl-lysine to validate the acetylation status of NSP4 protein in HEK293 cells.** A FLAG-tag NSP4 protein was expressed in human HEK293 cells and immunoprecipitated with FLAG-tag antibody. Anti-acetylated lysine and anti-FLAG tag antibodies were used to immunoblot the acetylation status of FLAG-NSP4 protein.

**Fig. 4:**
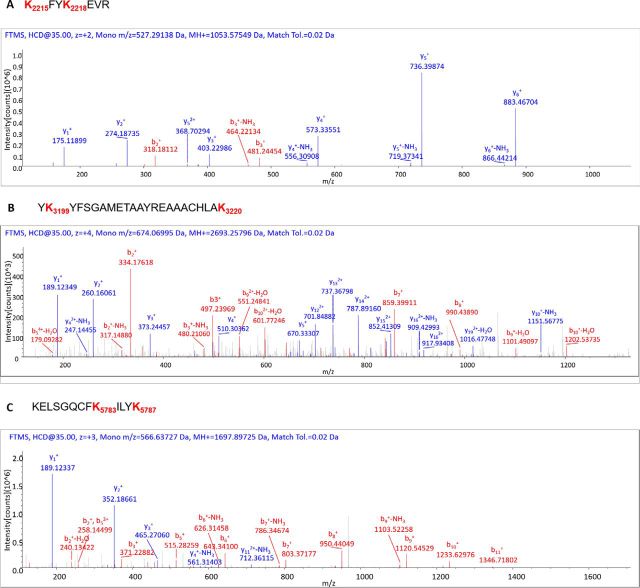
**MS2 spectrum of synthetic acetylated peptides.** MS/MS Spectra of synthetic acetyl-lysine containing peptides. *A*, **K_2215_**FY**K_2218_**EVR; *B*, Y**K_3199_**YFSGAMETAAYREAAACHLA**K_3220_**; *C*, KELSGQCF**K_5783_**ILY**K_5787_**.

To further confirm that the acetylation events identified was valid, we cloned the ORF NSP4 which includes the tentative SIRT1 substrate site K3220 and expressed it in HEK293 cells. NSP4 protein is one of the 15 cleavage products generated from pp1ab, which positioned ranging from 2741 to 3247 amino acid residue on pp1ab. With the FLAG tag on the expression vector, we were able to IP the NSP4 proteins and visualized its acetylation status with pan-acetyl-lysine antibody. As shown in Fig. 3, the NSP4 protein expressed in HEK293 cells was indeed acetylated significantly. The data confirmed our findings that pp1ab was acetylated in human cells. In addition, three synthetic acetylated peptides were obtained (Beijing SBS Genetech) and injected into Orbitrap MS machine to obtain MS^2^ spectrum (Fig. 4). The spectrums for the synthetic peptides indeed represented key fragment ions represented in our previous identification spectrums. We therefore concluded that the acetylation sites identified were reliable.

## DISCUSSION

**Fig. 5:**
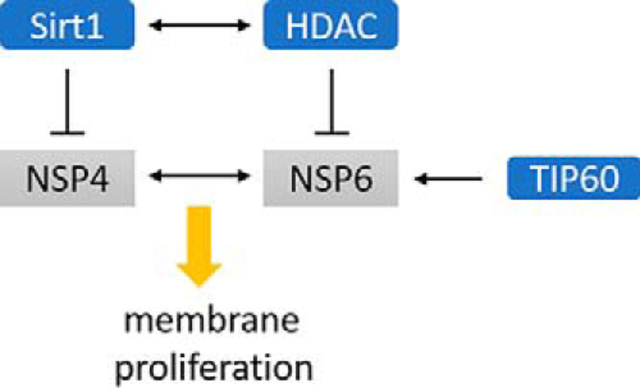
Potential interaction of upstream factors of acetylation events of pp1ab based on bioinformatic analysis.

In this study, acetylation sites on MERS-CoV replicase polyprotein pp1ab have been identified for the first time. The modified lysine sites are all highly conserved evolutionarily and therefore might be of crucial importance in viral adaptation to host environment. Bioinformatic analysis have suggested potential upstream factors that regulates certain acetylation sites, including SIRT1, HDAC, and TIP60 family members. Given that SIRT1 was reported as a proviral factor for MERS-CoV replication (22) and interacted with HDAC to regulate p53 activity (23, 24), which is known to suppress replication of another coronavirus SARS-CoV (25); MERS-CoV replication is plausibly regulated by acetylation through a complex network (Fig. 5). As NSP4 and NSP6 are known to interact with each other to form double membrane vesicles involved in coronaviral RNA replication (26), their acetylation might also be involved in the regulation of the MERS-CoV replication as well.

The identification and bioinformatic analysis of acetylation sites on MERS-CoV viral proteins would help to further understand the detailed molecular mechanism by which virus adapts to host environment to achieve optimal growth. The information obtained in the study also shed the light of a potential therapeutic target against MERS-CoV infection, as acetylation might be crucial in the host adaptation process of MERS-CoV virus. Further studies on how the acetylation site was regulated might help to dissect the actual role of NSP4 during MERS replication.

## DATA AVAILABILITY

Raw data and search result in .msf format has been deposit to Harvard Dataverse and available for reviewing at address: https://doi.org/10.7910/DVN/MTDOG7.
